# Probiotics in Nanotechnology-Driven Wound Healing: From Mechanistic Insight to Clinical Promise

**DOI:** 10.3390/pharmaceutics17070805

**Published:** 2025-06-21

**Authors:** Milind Umekar, Anis Ahmad Chaudhary, Monali Manghani, Supriya Shidhaye, Pratiksha Khajone, Jayashri Mahore, Hassan Ahmad Rudayni, Rashmi Trivedi

**Affiliations:** 1 Department of Pharmaceutics, Smt. Kishoritai Bhoyar College of Pharmacy, Kamptee, Nagpur 441002, Maharashtra, India; 2 Department of Biology, College of Science, Imam Mohammad Ibn Saud Islamic University (IMSIU), Riyadh 11623, Saudi Arabia; 3 Department of Quality Assurance, Smt. Kishoritai Bhoyar College of Pharmacy, Kamptee, Nagpur 441002, Maharashtra, India; 4 Department of Pharmaceutics, Sinhgad College of Pharmacy, Vadgaon (bk), Pune 411041, Maharashtra, India

**Keywords:** chronic wounds, probiotics, nanotechnology, wound healing, nanoformulations, drug delivery systems

## Abstract

Chronic wounds, including diabetic foot ulcers and pressure sores, are becoming more prevalent due to aging populations and increased metabolic problems. These wounds often persist due to impaired healing, chronic inflammation, oxidative stress, and infections caused by multidrug-resistant pathogens, making conventional treatments—including antibiotics and antiseptics—largely inadequate. This creates an urgent need for advanced, biologically responsive therapies that can both combat infection and promote tissue regeneration. Probiotics have surfaced as a viable option owing to their capacity to regulate immune responses, impede pathogenic biofilms, and generate antibacterial and antioxidant metabolites. However, their clinical application is limited by poor viability, sensitivity to environmental conditions, and short retention at wound sites. Nanotechnology-based delivery systems address these limitations by protecting probiotics from degradation, enhancing site-specific delivery, and enabling controlled, stimuli-responsive release. Encapsulation techniques using materials like chitosan, PLGA, liposomes, nanogels, nanofibers, and microneedles have shown significant success in improving wound healing outcomes in preclinical and clinical models. This review summarizes the current landscape of chronic wound challenges and presents recent advances in probiotic-loaded nanotechnologies. It explores various nano-delivery systems, their mechanisms of action, biological effects, and therapeutic outcomes, highlighting the synergy between probiotics and nanocarriers as a novel, multifaceted strategy for managing chronic wounds.

## 1. Introduction

Chronic wounds, including diabetic foot ulcers (DFUs), venous leg ulcers (VLUs), and pressure ulcers, pose significant public health issues, particularly for the elderly or individuals with diabetes mellitus [1]. Epidemiological research shows that chronic wounds impact approximately 2.21 out of every 1000 people in developed nations, while DFUs affect up to 25% of those with diabetes and are responsible for 85% of diabetes-related amputations globally [2]. In the United States, the financial impact of chronic wounds exceeds USD 25 billion annually [3]. According to estimates, between 1% and 2% of people worldwide will sustain a chronic wound at some time in their lives. Approximately 8.2 million people, irrespective of infection status, suffered wounds, according to a 2018 retrospective investigation. Acute and chronic wound care under Medicare was estimated to cost between USD 28.1 billion to USD 96.8 billion. Between 1.5 and 2 million people in Europe are thought to have either acute or chronic wounds [4].

Chronic wounds are often marked by prolonged and excessive inflammation, hindered angiogenesis, microbial imbalance, and the formation of pathogen biofilms [5]. It is estimated that biofilms are present in approximately 78% of wounds that do not heal, providing a refuge for pathogens such as *Staphylococcus aureus*, *Enterococcus faecalis*, *Escherichia coli*, *Pseudomonas aeruginosa*, *Klebsiella pneumoniae*, and *Acinetobacter baumannii*, where they remain protected from the patient’s immune defenses and medical treatments. The global increase in antimicrobial resistance (AMR), particularly multi-drug resistance (MDR), worsens the situation, as the vast majority of chronic wound infections are caused by these resistant strains [6,7].

Although traditional antibiotic treatments have been central to managing wound infections, they come with several drawbacks [8]. Antibiotic overuse and improper dosing can also result in systemic adverse effects and delayed recovery because of collateral tissue damage [9]. In contrast, probiotics could serve as a useful supplement or alternative for managing chronic wounds [10,11].

Probiotics as a therapeutic option often face challenges due to their sensitivity to oxygen, heat, and the gastrointestinal environment, as well as their limited viability and retention at wound sites [12]. To address these issues, the use of nanotechnology for delivering probiotics has been suggested as an effective approach to enhance their stability, bioavailability, and targeted delivery [13].

Nanocarriers, such as liposomes, nanoparticles, polymeric hydrogels, and electrospun nanofibers, offer advantages like controlled release rates, protection against external degradation, and strong adhesion to moist wound surfaces [14,15]. In 2024, murine models infected with *S. aureus* showed 85% complete wound closure within a week after using a *Lactobacillus* (formerly designated *Lactobacillus* genus) microneedle patch, outperforming standard topical antibiotics [16]. Similarly, chitosan nanoparticles loaded with cell-free supernatants of *Lacticaseibacillus rhamnosus* (formerly designated as *Lactobacillus rhamnosus*) achieved over 95% inhibition of both mono- and dual-species biofilms without inducing resistance in vitro [17]. While previous reviews have either focused on probiotics [18] or nanotechnology-based approaches individually in the context of wound healing, or discussed their therapeutic benefits in broader terms, only a limited number have thoroughly addressed their integrated use for chronic wound management [19]. For instance, the wound-healing potential of probiotics and how nanotechnology may enhance their delivery and efficacy has been discussed in [20]. However, a dedicated review that critically evaluates the synergistic application of probiotics and nanotechnology—particularly through novel delivery platforms such as hydrogels, microneedles, or electrospun fibers—remains scarce [21]. This review therefore aims to bridge that gap by comprehensively exploring the mechanistic pathways, preclinical and clinical advances, and translational challenges of using probiotic-based nanotherapeutics for chronic wound healing.

Additionally, probiotics, extracellular vesicles, and smart biosensor-assisted probiotic dressings are emerging therapeutic approaches in regenerative medicine, yet they remain relatively unfamiliar. In this review, we critically examine the availability of probiotics as agents for wound healing and their integration with nanotechnology [22]. This combination of probiotics and nanotechnology offers a novel and potentially intricate pathway to address the limitations of existing wound therapies and to provide solutions for the increasing population with MDR-associated chronic wounds [23].

## 2. Pathophysiology of Chronic Wounds

Hemostasis, inflammation, proliferation, and remodeling are all components of the structured biological process that is wound healing. This process leads to successful tissue regeneration for acute wounds. On the other hand, chronic wounds—like DFUs, pressure ulcers, and VLUs—show reduced capacity to heal, mostly because of prolonged inflammatory phases, microbial colonization, and problems with angiogenesis [24]. Chronic wounds pose serious health risks and last longer than three months, especially for people with diabetes, vascular insufficiency, or restricted movement [25].

The overproduction of pro-inflammatory cytokines, such as TNF-α, IL-1β, and IL-6, together with increased levels of matrix metalloproteinases (MMPs) and reactive oxygen species (ROS), are characteristics of chronic wounds. Uncontrolled MMP production leads to extracellular matrix degradation and growth factor activity inhibition, whereas ROS-induced damage impairs fibroblast migration, keratinocyte proliferation, and endothelial function [26,27]. A fundamental factor in the persistence of wounds that do not heal is oxidative stress. Because of mitochondrial dysfunction, reactive oxygen species (ROS) generation and the creation of advanced glycation end-products (AGEs) are elevated in hyperglycemic situations, especially in diabetic wounds [28]. The accumulation of AGEs further exacerbates wound healing by promoting inflammation, impairing collagen synthesis, and disrupting cellular signaling pathways [29]. Elevated AGEs have been shown to enhance the formation of cross-links in extracellular matrix proteins, leading to the stiffening of the wound bed, which delays tissue remodeling and prolongs the chronicity of wounds [30]. Diabetic mice exhibited approximately a 2.7-fold increase in ROS compared to control groups, along with significantly reduced VEGF expression and epithelialization, *p* < 0.01 for both. These findings align with clinical evidence showing reduced neovascularization and less granulation tissue in biopsies from diabetic ulcers [31].

The presence of bacteria, frequently arranged in biofilms—dense collections of microorganisms encased in an extracellular matrix that generates itself—defines chronic wounds [32]. Biofilms are detected in about 78% of chronic wound samples, significantly hindering immune system clearance and increasing antibiotic tolerance by over 1000 times [33]. The biofilm’s matrix facilitates gene exchange, including that which causes antibiotic resistance, and serves as a barrier against antimicrobial drugs [34]. *Staphylococcus epidermidis* is among the most concerning pathogens, typically a harmless organism but increasingly prevalent as an infectious agent in chronic wounds due to its biofilm-forming ability and genetic adaptability [35]. In a metagenomic analysis, it was discovered that *S. epidermidis* strains from venous leg ulcers that expressed virulence genes linked to adhesion (icaA, aap), biofilm persistence, and antimicrobial resistance (mecA, mupA) [19]. These *S. epidermidis* strains triggered elevated levels of IL-1β and IL-8 in ex vivo human wounds and were linked to non-healing wounds in 83% of patients [36].

Chronic wounds are linked to significant dysbiosis in the microbiome, marked by reduced microbial diversity and an abundance of harmful bacteria like *P. aeruginosa*, *E. faecalis,* and *S. aureus* [37]. There is a notable decline in beneficial bacteria such as *Cutibacterium acnes* and various species of formerly designated *Lactobacillus* like *Lentilactobacillus crispatus*, which play roles in maintaining the skin barrier and modulating the immune system. This reduction in microbial presence alters the local equilibrium, undermines the interaction between host keratinocytes and immune cells, disrupts the host’s antimicrobial capabilities, and results in persistent inflammation [38].

The importance of the skin microbiota in regulating local immunity is now generally acknowledged. Commensal organisms can boost the production of antimicrobial peptides (AMPs), improve the integrity of epithelial tight junctions, and affect toll-like receptor (TLR) signaling [39]. In the absence of regulation, the wound bed can become overrun by polymicrobial communities, leading to the activation of immune tolerance and a reduced neutrophil response, as seen in chronic wounds with dual-species biofilms [40].

Chronic wounds are caused by a complex disruption in immune regulation, microbial equilibrium, and oxidative balance. This leads to a persistently inflamed microenvironment characterized by microbial biofilms and cellular aging, which creates a niche resistant to conventional treatments [5]. These complexities highlight the need for multimodal interventions that can potentially restore the microbiome, regulate immune responses, and overcome resistance mechanisms [41]. The combined use of probiotics and nanotechnology, discussed in later sections, offers a promising strategy to tackle these interconnected pathophysiological challenges through a mechanism-based and targeted therapeutic approach [42].

## 3. Role of Probiotics in Wound Healing

**Figure 1:**
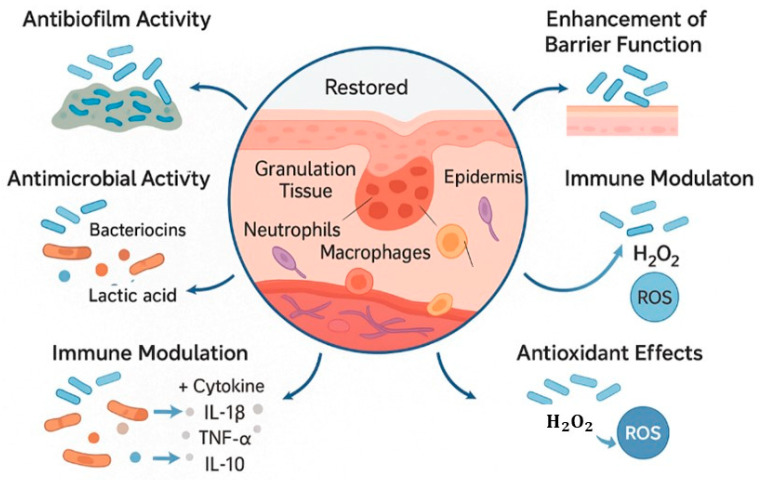
Multifactorial roles of probiotics in wound healing; Antimicrobial action: via bacteriocins, organic acids, and hydrogen peroxide; Immunomodulation: regulating cytokines, macrophage polarization, dendritic cell activity; Oxidative stress reduction: through decreased ROS generation; Barrier enhancement: via upregulation of tight junction proteins; Tissue regeneration: through fibroblast activation and angiogenesis (Figure Created in Biorender. Monali Manghani (2025) https://app.biorender.com/illustrations/67a2f3345763bcd775f37436?slideId=70b8619c-8934-4282-b450-b3043be5b055, accessed on 17 June 2025).

Recent studies have illuminated the role of probiotics in promoting wound healing. For instance, probiotics exhibit antimicrobial effects by producing substances like bacteriocins, organic acids such as lactic and acetic acids, and hydrogen peroxide, which inhibit the growth of harmful pathogens. They also impact the immune system by influencing cytokine production, changing macrophage polarization, and adjusting dendritic cell function, thereby reducing inflammation, as shown in Figure 1. Furthermore, probiotics reduce reactive oxygen species (ROS) levels to lessen oxidative stress and improve the epithelial barrier by increasing the production of tight junction proteins. Furthermore, they stimulate fibroblasts and encourage the production of new blood vessels, which aids in the growth of granulation tissue [43,44].

A significant study from 2022 revealed that applying *Lactiplantibacillus plantarum* (formerly designated as *Lactobacillus plantarum*) topically to skin wounds in mice resulted in a 42% reduction in wound size by the seventh day compared to the control group (*p* < 0.01) [45]. In the wound microenvironment, this result was associated with a decrease in neutrophil infiltration and an increase in modifying growth factor-beta 1 (TGF-β1) and vascular endothelial growth factor (VEGF) levels [46]. Additional research has underscored the importance of *L. plantarum* in healing diabetic wounds. In vivo studies using diabetic rat models shown that *L. plantarum* facilitates wound healing by regulating inflammation, namely by preventing the activation of the NLRP3 inflammasome and pyroptosis pathways brought on by AGEs. Increased re-epithelialization and tissue regeneration are the outcomes of this modification [47].

Innovative formulations that combine *L. plantarum* with other substances have demonstrated synergistic effects. For instance, research on a honey—*L. plantarum* mixture revealed that this combination effectively inhibited the growth of harmful bacteria like *S. aureus*, *P. aeruginosa*, and *E. coli* [48]. This formulation disrupted biofilm formation and reduced the expression of quorum sensing genes, thereby boosting antibacterial activity and aiding wound healing in infected rat models. Progress in probiotic-based wound care has also resulted in the creation of new delivery systems [49]. A study conducted in 2025 introduced an in-situ gelling formulation aimed at preserving probiotics for wound healing purposes [50]. This formulation supports the continuous production of antimicrobial agents by metabolizing added glycerol, thus speeding up the healing process of infected wounds [51].

Research continues to advance in demonstrating the benefits of probiotics in clinical environments, applicable to both animal and human studies [52]. Diabetic rats receiving oral supplementation with *Lacticaseibacillus casei* (formerly designated as *Lactobacillus casei*) experienced faster wound contraction, a 2.3-fold increase in type I collagen deposition, and enhanced histological wound scores by the 10th day of recovery (*p* < 0.01) [53]. Additionally, in a comparable model, the oral administration of *L. rhamnosus GG* was found to restore angiogenic signaling in wounds, evidenced by elevated expression of HIF-1α and mRNA expression of VEGF [54].

Probiotics applied topically have produced significant clinical evidence supporting their effectiveness. In a review that examined both human and animal studies, it was concluded that the topical use of *Lactobacillus* strains led to improved re-epithelialization and decrease in bacterial colonization, with no immune reactions observed in the vast majority (over 90%) of the cases studied [18].

Probiotic therapy’s capacity to affect immune responses in chronic wounds is one of its main advantages. By increasing anti-inflammatory substances like IL-10 and decreasing pro-inflammatory cytokines, probiotics aid in the reduction of inflammation. Furthermore, they encourage macrophages to change from the M1 to the M2 phenotype, which is crucial for reducing inflammation and promoting tissue healing [55].

*Lactobacillus* spp. function as immune modulators in diabetic foot ulcers by reducing IL-1β and TNF-α levels while also reestablishing TLR2/4 balance in keratinocytes. Biofilm development on chronic wounds frequently plays a significant role in hindering the healing process [56]. Probiotics are known for their strong antibiofilm properties. Cell-free supernatants derived from *L. rhamnosus GG* were able to inhibit the formation of biofilms by *S. aureus* and *P. aeruginosa* by more than 95%, without causing resistance even after prolonged exposure [57,58]. Beyond preventing biofilm formation, these supernatants also lowered the levels of quorum sensing associated with biofilms and reduced the expression of biofilm-related genes (icaA and lasR), indicating a promising alternative for addressing persistent biofilm infections without the use of conventional antibiotics [59].

*Limosilactobacillus reuteri* (formerly known as *Lactobacillus reuteri*) is another probiotic that shows promise due to its production of reuterin and other antimicrobial peptides (AMPs), which can selectively target wound pathogens while sparing beneficial skin microbiota [60]. This targeted antimicrobial effect of probiotics allows for the specific treatment of wound pathogens, unlike antibiotics, which often act in a non-specific way. Probiotics do not cause further dysbiosis, a problem associated with broad-spectrum antibiotics that can delay the wound healing process [12].

Recent research suggests that postbiotics, such as bacterial lysates, metabolites, and heat-killed strains, along with Extracellular Vesicles (EVs), might offer probiotic-like benefits with greater stability. For instance, EVs derived from *L. plantarum* have been found to enhance keratinocyte migration and reduce MMP-9 expression in vitro, promoting wound healing without the risk associated with live cell delivery [61,62].

### 3.1. Mechanism of Action of Probiotics Against Pathogens with Specific Strains

Probiotics help shield the host from harmful infections, particularly when it comes to chronic wounds, by acting as antimicrobials through a variety of ways. Although these mechanisms depend on strain, they frequently include several complementary actions [63].

The synthesis of antimicrobial compounds is one of the main ways probiotics fight against infections. Bacteriocins, organic acids (such lactic and acetic acids), and hydrogen peroxide are produced by strains such as *L. rhamnosus GG* and *L. plantarum*, which lower pH and damage the integrity of dangerous pathogen cells [64,65]. Probiotics are an efficient treatment option for preventing infection in chronic wounds because of these compounds, which stop the growth of bacteria like *S. aureus* and *E. coli* [66].

In addition to antimicrobial production, probiotics also support the immune system by modulating the host’s immune response. They influence cytokine production, alter macrophage polarization, and adjust dendritic cell activity [67]. For example, *L. plantarum* and *Bifidobacterium longum* can modulate the immune response, which helps reduce inflammation and promotes pathogen clearance. Additionally, by boosting the expression of tight junction proteins, which are essential for preserving the integrity of the skin or gut lining, probiotics such as *L. casei* and *L. rhamnosus GG* aid in strengthening the epithelial barrier. This lowers the chance of infection by making it more difficult for pathogens to infiltrate [68,69].

Probiotics such as *L. plantarum* and *L. rhamnosus GG* can increase the release of anti-inflammatory cytokines like IL-10 while decreasing the production of pro-inflammatory cytokines like TNF-α, IL-6, and IL-1β. Because it aids in the resolution of the inflammatory phase and encourages tissue regeneration, this anti-inflammatory impact is essential for chronic wound healing [70].

Probiotics play an essential role in restoring microbial homeostasis. By outcompeting harmful pathogens and fostering beneficial bacteria, probiotics help maintain a balanced microbial ecosystem on the skin or within the gut, which is crucial for healing chronic wounds. *Lactobacillus* spp. (formerly designated *Lactobacillus* genus) and *Bifidobacterium* spp. contribute to this balance, improving resistance to infection and supporting the overall healing process [71].

### 3.2. Clinical Evidence and Human Trials

A preliminary study by Stuermer et al. investigated the effects of oral multispecies probiotics, including *L. plantarum*, *L. rhamnosus*, and *Bifidobacterium bifidum*, on 20 diabetic patients with chronic, difficult-to-heal wounds. These patients, who lived in a long-term care facility, took the probiotics for a duration of six months. Out of the 20 participants, only 13 completed the study, and all remained quite ill. Nevertheless, among these 13 individuals, 5 experienced complete wound closure, and most reported improvements in a wound-specific quality of life measure, particularly in terms of pain and mobility. The study also noted a simultaneous decrease in the microbial load of *S. aureus* and *P. aeruginosa*, along with a partial restoration of commensal populations following the use of probiotics [72].

Numerous studies have reported similar results in non-diabetic patients. For instance, a randomized trial conducted in 2023 found that applying *Limosilactobacillus fermentum* (formerly known as *Lactobacillus fermentum*) topically to burn wounds reduced healing time by 21% compared to silver sulfadiazine (*p* < 0.05), while systemic treatment led to a decrease in local cytokines IL-6 and TNF-α [73]. Table 1 provides a summary of key preclinical and clinical studies examining the impact of various probiotic strains on wound healing. It outlines the delivery methods, mechanisms of action, and significant outcomes observed in different models.

## 4. Nanotechnology in Wound Healing and Probiotic Delivery

Nanotechnology focuses on creating materials that are less than 100 nm in size, which allows for high surface-area-to-volume ratios, precise therapeutic delivery, and controlled-release mechanisms. These features are particularly effective in tackling the complex nature of chronic wounds, which often require managing infection, reducing inflammation, and promoting tissue regeneration simultaneously [79]. Researchers have explored various nanomaterials for their potential in wound treatment, including polymeric nanoparticles like chitosan and PLGA, lipid-based carriers such as solid lipid nanoparticles, liposomes, and inorganic nanoparticles like silver, zinc oxide and gold [80].

**Figure 2:**
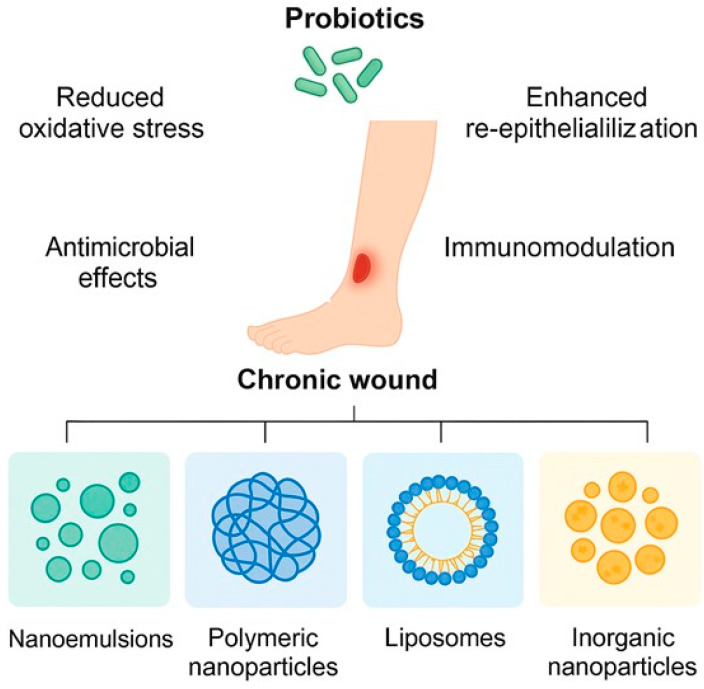
Nanotechnology-based encapsulation strategies for enhancing probiotic stability in wound healing applications (Figure Created in Biorender. Monali Manghani (2025) https://app.biorender.com/illustrations/678de110f49fd8be43cad043?slideId=3e28ea1f-4dd3-4295-85fa-2be90ac40d86, accessed on 17 June 2025).

To maintain the viability of probiotics in challenging biological settings, various nanoencapsulation methods have been devised, as shown in Figure 2. These methods encompass nanoemulsions, polyelectrolyte complexation, spray-dried or freeze-dried nanobeads, and electrospun nanofibers [81].

**Nanoemulsions** are thermodynamically stable colloidal systems formed by dispersing one liquid phase into another (typically oil-in-water) using surfactants, resulting in droplets ranging from 20 to 200 nm. These systems protect probiotic cells by encapsulating them in the dispersed phase, thus shielding them from oxidative degradation, acidic environments, and enzymatic hydrolysis. Their small droplet size ensures enhanced mucosal or dermal penetration, while the high surface-area-to-volume ratio promotes sustained and localized release of viable microorganisms. In the context of wound healing, probiotic-loaded nanoemulsions have demonstrated accelerated wound closure, reduced pathogen colonization, and improved re-epithelialization in preclinical models [82,83,84].

**Spray-dried and freeze-dried nanobeads** represent another widely employed method for improving probiotic stability. A probiotic-polymer solution is compressed into a hot air stream during the spray drying process, which quickly evaporates the solvent and creates solid nanobeads. In contrast, freeze drying preserves cellular viability and structure by sublimating frozen aqueous probiotic compositions under low pressure. At room temperature, the resultant dry powders are very stable and can be rehydrated as needed [85,86]. These nanobeads are ideal for incorporation into hydrogels, ointments, or dry wound dressings. Studies have shown that probiotics preserved using these techniques retain over 80–90% viability for several months and exhibit effective immunomodulatory and antimicrobial actions when reintroduced into wound environments [87].

**Electrospun nanofibers** provide a sophisticated platform that mimics the extracellular matrix of human skin, providing both structural support and controlled probiotic release. In this technique, probiotic-laden polymer solutions—composed of biodegradable materials like gelatin, polycaprolactone (PCL), or polyvinyl alcohol (PVA)—are subjected to high-voltage electric fields to form ultrafine fibers collected into porous mats. These nanofibers offer high porosity and surface area, promoting gas exchange, moisture retention, and cell migration—all critical elements in wound healing [88]. Embedded probiotics within the fibers are protected from desiccation, UV exposure, and mechanical damage, while sustained release over time ensures prolonged microbial activity. Recent research has shown that electrospun nanofibers loaded with *L. plantarum* significantly improved healing outcomes in diabetic wound models, with enhanced granulation tissue formation and reduction of biofilm-forming pathogens [89].

Recent studies have demonstrated that encapsulation of *L. plantarum* using biopolymers such as chitosan, alginate, and gelatin significantly enhances its viability and stability. For instance, *L. plantarum* encapsulated within alginate-gelatin or transglutaminase-crosslinked gelatin hydrogels showed improved resistance to gastrointestinal stress and prolonged storage stability, maintaining high viability for up to 28 days at refrigerated conditions [90]. These encapsulation strategies have proven effective in preserving probiotic functionality and supporting their therapeutic potential in wound healing applications [91].

A novel approach involved encapsulating *L. reuteri* in PLGA nanoparticles, enabling a sustained release over 72 h. In vivo experiments revealed that this formulation notably reduced the density of *S. aureus* biofilms in wounds with active infections. These examples underscore how nanotechnology, particularly through the encapsulation of probiotics, is revolutionizing wound care by enhancing the stability, efficacy, and targeted delivery of bioactive compounds [91].

### 4.1. Advanced Probiotic-Nano Systems

Recent advancements have transitioned from basic encapsulation to bioresponsive and multifunctional systems. Microneedle (MN) patches containing live *L. rhamnosus* GG outperformed traditional antibiotic treatments for *S. aureus*-infected wounds in mice, with 85% of treated cases fully healing within seven days, demonstrating remarkable safety and effectiveness. Even after 60 days of cold storage, these MN patches maintained over 80% bacterial viability [92].

Unlike live probiotics, postbiotics—comprising inactivated bacterial cells, metabolites, and cell-free supernatants—are more chemically stable and pose no risk of microbial overgrowth [93]. Recent studies have focused on encapsulating these bioactive molecules within nanocarriers to enhance their therapeutic delivery. One study involved cell-free extracts of *L. fermentum* encapsulated in a hydrogel with zinc oxide nanoparticles, resulting in a 2.4-fold increase in re-epithelialization compared to the control and a 48% reduction in IL-1β concentration in burn models [94]. Stimuli-responsive carriers, such as pH or ROS-sensitive nanogels, release probiotics or their bioactive components within the local wound environment. In 2023, researchers developed a glucose-responsive delivery system for *L. acidophilus* that released hydrogen peroxide in hyperglycemic wounds, promoting angiogenesis and reducing bacterial load [95].

#### 4.1.1. Chitosan Nanoparticles

Because of its inherent antibacterial, biodegradable, and biocompatible qualities, chitosan—a naturally occurring polysaccharide produced from chitin—has attracted a lot of interest in wound healing applications. Chitosan’s cationic properties enable it to interact with negatively charged microbial cell membranes, increasing its antibacterial effectiveness [96,97]. Moreover, chitosan promotes hemostasis and supports cellular proliferation and migration, essential for tissue regeneration. Nanoparticulate chitosan exhibits a higher surface area-to-volume ratio, facilitating better interaction with biological tissues and more efficient drug delivery [98]. In one study, chitosan nanoparticles containing the antibiotic cefepime were created and incorporated into hydrogel membranes. In rat models, these membranes showed considerable antibacterial action against common wound pathogens, controlled medication release, and hastened wound closure [99].

Chitosan, despite its biocompatibility and mucoadhesiveness, faces significant practical limitations that restrict its use as the main encapsulating matrix. First, its poor solubility at neutral-to-alkaline pH remains a major challenge. Even with chemical modifications like N-methylene phosphonic acid chitosan (NMPC-GLU) improving solubility, these derivatives are still being developed and lack extensive validation in primary encapsulation systems [100]. Second, mechanical instability is widely reported. A 2022 review noted that chitosan microparticles required multiple layers—such as pectin–chitosan–collagen—to maintain structural integrity, indicating its insufficiency when used alone. Third, limited drug loading and burst release issues are repeatedly observed. For example, a 2022 study on ghrelin-loaded chitosan liposomes highlighted that PLGA outperformed chitosan-based carriers in encapsulation efficiency, sustained release, and protection against enzymatic degradation [101]. Fourth, stability challenges in physiological environments persist: pulmonary delivery systems doped with chitosan exhibit variable performance and depend heavily on synthetic modifiers to stabilize drug delivery. Last, despite frequent use in food-grade encapsulation (e.g., essential oils), chitosan-based nanocarriers continue to face reproducibility and scalability concerns, even in a well-controlled 2022 study [102].

#### 4.1.2. PLGA Nanoparticles

A biodegradable and biocompatible copolymer, poly(lactic-*co*-glycolic acid) (PLGA) is widely used in drug delivery systems because of its excellent breakdown kinetics and capacity to encapsulate a variety of therapeutic compounds [103]. PLGA nanoparticles have been used in wound healing applications to improve therapeutic outcomes by enhancing the stability, controlled release, and targeted delivery of bioactive chemicals. The effectiveness of PLGA nanoparticles in delivering growth factors to support angiogenesis and tissue regeneration has been shown in recent investigations [104]. For example, a study created PLGA nanoparticles that co-encapsulated the genes for vascular endothelial growth factor A (VEGFA) and basic fibroblast growth factor (bFGF) [105]. Rats with deep burn wounds treated with these nanoparticles showed improved granulation tissue development, faster wound healing, and more epithelialization than controls [106].

In a different study, PLGA nanospheres loaded with curcumin were created and incorporated into a topical nanogel. In Wistar rats, this formulation demonstrated strong anti-inflammatory properties and accelerated the healing of cutaneous wounds, demonstrating the potential of PLGA nanoparticles to deliver phytochemicals for skin regeneration [107]. Furthermore, Ref. [108] synthesized polymeric curcumin nanospheres using PLGA, which demonstrated potent antibacterial activity and effectively inhibited lysozyme aggregation. These nanospheres facilitated wound healing in *Drosophila melanogaster* models, suggesting their applicability in managing microbial infections in wounds.

#### 4.1.3. Nanofibers

Because of their structural resemblance to the extracellular matrix (ECM), high surface-area-to-volume ratio, and ability to transport drugs continuously, nanofibers—which are usually made by electrospinning—have become extremely promising scaffolding materials for wound healing [109]. These features allow nanofibers to promote cellular adhesion, proliferation, and migration while offering excellent control over the local wound microenvironment. Electrospun nanofibers can effectively encapsulate probiotics and maintain their bioactivity [110]. For example, Ref. [111] developed polycaprolactone (PCL)/gelatin nanofibers loaded with *L. rhamnosus GG*, achieving over 70% bacterial viability over a 10-day period while significantly improving the tensile strength and epithelial regeneration in rat wound models.

Recent investigations further validate this approach. Xu et al. successfully prepared pectin/PVA nanofibers encapsulating *L. rhamnosus* 1.0320 via electrospinning. The resulting fibers demonstrated strong preservation of bacterial viability—89.65% immediately after fabrication and 85.32% after 21 days of storage at 4 °C—confirming the matrix’s capacity to maintain long-term functionality of probiotics in dry formulation [112]. In another study, Stojanov et al. developed PEO-based nanofibers incorporating genetically engineered vaginal *Lactobacillus* (formerly designated *Lactobacillus* genus) strains. These fibers showed rapid dissolution (complete in 30–45 min), high bacterial viability, and no hemolytic or cytotoxic effects. Moreover, the fluorescent and luminescent markers allowed real-time tracking of bacterial adhesion to Caco-2 epithelial cells, with adhesion rates ranging from 0.5 to 50% depending on the strain. These findings highlight both the safety and precision of nanofiber-based probiotic delivery systems for mucosal and wound applications [113].

Further, hybrid nanofiber systems have been explored for co-delivery of probiotics with growth factors or postbiotics [114]. The versatility of nanofiber compositions—ranging from synthetic polymers like PCL and polylactic acid (PLA) to natural biopolymers such as gelatin and collagen—has enabled researchers to tailor wound dressings to specific wound types and healing phases. Some designs also incorporate layered or core–shell architectures, allowing sequential release of bioactives [115]. Nanofibers represent a sophisticated and flexible platform for the topical delivery of probiotics and other wound-healing agents. Their ability to mimic native tissue architecture while enabling localized and sustained therapeutic delivery makes them ideal for chronic and infected wound management [116].

#### 4.1.4. Nanobeads

Nanobeads are spherical, nano-sized particles commonly fabricated from biodegradable polymers and are used to encapsulate bioactive compounds, including probiotics like *L. reuteri*, *L. fermentum*, and *Bacillus subtilis*, enzymes like NanoLuc luciferase, and postbiotics like organic acids and *reuterin* [117]. These structures are advantageous due to their uniform morphology, high encapsulation efficiency, and ability to protect labile biological agents from harsh environmental conditions such as gastric pH, oxidative stress, and temperature fluctuations. In the context of wound healing, nanobeads serve as effective delivery systems that prolong the bioactivity of encapsulated compounds at the wound site [118].

#### 4.1.5. Microneedles

To provide bioactive substances painlessly straight into the dermal layer, microneedles (MNs) are a minimally invasive and highly targeted delivery system that can penetrate the stratum corneum without entering deeper nerve-rich regions [119]. Adding probiotics or their bioactive derivatives to microneedle systems has become a novel way to speed up wound healing in recent years, particularly for chronic or infected wounds [120].

One of the most significant advancements was reported by [120], who developed a biodegradable microneedle patch encapsulating live *L. rhamnosus GG* within a polyvinyl alcohol (PVA)/hyaluronic acid matrix. When applied to *S. aureus*-infected wounds in mice, this system achieved complete wound closure in 85% of cases within seven days—markedly outperforming traditional topical antibiotics. Importantly, the MNs retained over 80% bacterial viability after 60 days of cold storage, underscoring their long-term stability.

The capacity of probiotic-loaded MNs to introduce live bacteria straight into the wound bed, where they can influence immune responses, promote re-epithelialization, and have localized antimicrobial effects, is the mechanism underlying their effectiveness. Specifically, it is known that the LGG strain reduces local inflammation by enhancing regulatory T-cell responses and secreting antimicrobial peptides [54].

#### 4.1.6. Postbiotic Encapsulation

Postbiotic-loaded nanogels, which deliver bacterial metabolites instead of live bacteria, have been evaluated in clinical models. Postbiotic-based nanodelivery systems have emerged as a distinct and promising strategy in wound care [121]. Recent studies have emphasized the importance of encapsulating postbiotics within nanocarriers to protect them from degradation, prolong their bioavailability, and enable targeted release at the wound site [122]. In a rat burn model, this postbiotic formulation accelerated wound closure by 2.4-fold compared to the control and significantly decreased pro-inflammatory IL-1β levels by 48%, suggesting strong immunomodulatory effects [123].

#### 4.1.7. Other Nanocarriers and Emerging Approaches

In addition to well-studied platforms like chitosan, PLGA, hydrogels, nanofibers, nanobeads, microneedles, and postbiotic systems, a range of emerging nanocarriers is being explored to overcome the limitations of conventional wound care and probiotic delivery. These systems emphasize improved stability, site-specific targeting, and multifunctional therapeutic performance.

**Lipid-based nanocarriers**, such as liposomes and solid lipid nanoparticles (SLNs), have demonstrated potential for encapsulating both live probiotics and postbiotic components. A recent study by [124] designed liposome-encapsulated *L. rhamnosus GG*, showing prolonged bacterial viability and enhanced adhesion to keratinocytes in vitro. When tested in a diabetic wound model, these liposomes facilitated a 1.8-fold acceleration in epithelial regeneration and reduced TNF-α expression by 45%, confirming their dual anti-inflammatory and pro-healing function.

**Dendrimers**—branched, tree-like polymers—are another emerging system with high loading capacity and surface functionalization potential. Although still in the preclinical phase, Patel et al. formulated cationic poly(amidoamine) (PAMAM) dendrimers to deliver short peptides derived from probiotic secretomes. These constructs exhibited selective antimicrobial activity against *P. aeruginosa* while sparing commensal skin flora and significantly promoted angiogenesis in wound scratch assays [125,126].

**Metal-organic frameworks (MOFs)** have recently emerged as highly tunable platforms with large surface areas, ideal for pH-responsive or enzyme-triggered release of therapeutic payloads. Furthermore, the capacity of nanoclays and bioinorganic hybrids to adsorb probiotic metabolites or cytokines and alter wound pH or oxidative stress has been studied [127].

**Self-assembling peptide nanostructures**, such as RADA16, have also been explored as scaffolds to deliver probiotic lysates or metabolites. These biomaterials create an electroactive, nanofibrous matrix that mimics the ECM and promotes fibroblast migration. Research by [128] demonstrated enhanced collagen I expression and lower bacterial load when postbiotic-loaded RADA16 gels were applied to chronic wounds.

These novel nanoplatforms expand the landscape of wound therapeutics by combining structural support with bioactive signaling, controlled release, and improved biocompatibility. As more advanced designs are validated through in vivo and clinical models, they hold great promise for integration into next-generation bioactive dressings and personalized wound management strategies.

## 5. Probiotic–Nanotechnology Synergy in Chronic Wound Healing

**Figure 3:**
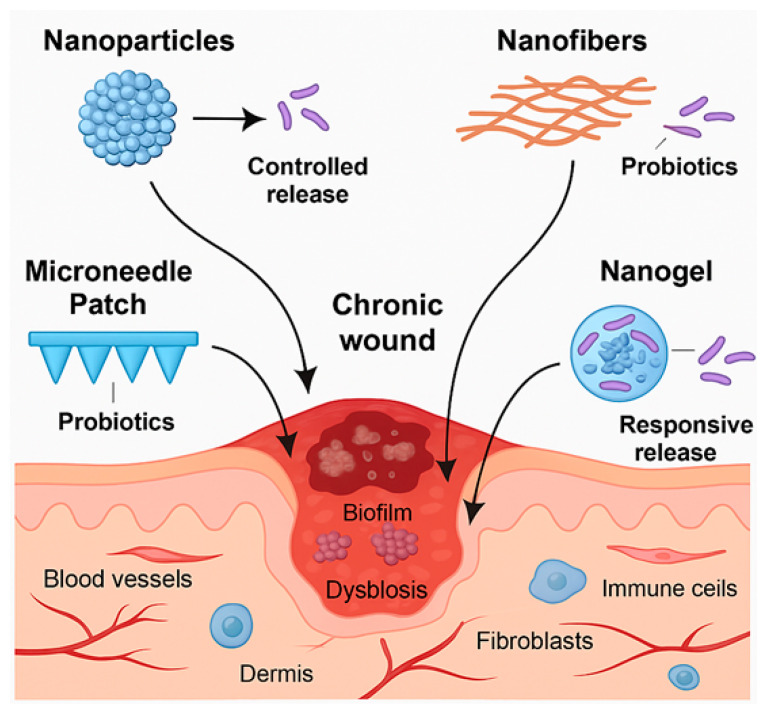
Synergistic mechanisms of probiotic–nanotechnology hybrids in chronic wound healing. (Figure Created in Biorender. Monali Manghani (2025) https://app.biorender.com/illustrations/67a1a45f46c7e641660b8370?slideId=a8d17636-0ae1-4727-bb56-54f6e4d94af8, accessed on 17 June 2025).

A promising development in the management of chronic wounds is the use of probiotics augmented with nanotechnology. In addition to offering environmental protection and structural support, nanoencapsulation improves retention and delivers more efficient functionality. This combination minimizes issues with degradation in oxidative microenvironments, retention at the wound site, and survivability while providing biological benefits [129]. Various encapsulation techniques, such as using chitosan nanoparticles, liposomes, and nanogels, present different mechanistic strategies. Figure 3 illustrates the synergistic mechanisms through which probiotic–nanotechnology hybrids contribute to chronic wound healing. Chitosan-encapsulated *L. plantarum* achieved 93% encapsulation efficiency, significantly enhancing wound healing in diabetic rats treated with the encapsulated bacteria compared to a non-encapsulated strain, with a notable increase in collagen deposition and granulation tissue formation (*p* < 0.01) [130].

Utilizing nanotechnology for delivering probiotics to wound sites offers advantages such as enhanced environmental resistance and controlled release kinetics. According to [131], nanobeads containing *L. rhamnosus GG* retained 90% of probiotic viability after 90 days in ambient storage conditions. However, a decline of over 60% was observed when probiotics were freeze-dried. Delivery systems that react to glucose, ROS, or pH enable targeted release. Huang et al. created a glucose-responsive hydrogel with *L. reuteri* for H_2_O_2_ release, which reduced wound bacteria by more than 90% and improved epithelial regeneration in diabetic mouse models. This was due to the glucose-enhanced, hydrogen peroxide-producing strain, or the increased metabolic effect on bacteria by glucose [132,133].

Microneedle array platforms offer opportunities for delivering viable probiotics in a less invasive and more localized manner. Two studies [12,134] developed microneedles infused with *L. rhamnosus GG*, achieving over 85% healing in *S. aureus* wound infections within a week. The formulation maintained over 80% of viable bacteria after being stored in a structured refrigerated environment for two months, highlighting its potential for long-term use.

Beyond their antimicrobial and regenerative capabilities, probiotics also modify the immune microenvironment of chronic wounds, which are often plagued by persistent inflammation marked by a predominance of M1 macrophages and elevated levels of IL-6, IL-1β, and TNF-α [135,136]. In a study, it was demonstrated that nanogel-loaded *L. fermentum* significantly impacted (*p* < 0.01) the immune microenvironment in a diabetic wound model by increasing M2 macrophage populations (CD206+ cells) by 2.6-fold and reducing IL-6 and TNF-α levels. By blocking the NF-κB signaling pathway and reviving antioxidant enzymes like catalase and superoxide dismutase, the administration of nanoformulated probiotics decreased oxidative stress [137].

Probiotic-nano hybrids, as a comprehensive solution, offer a bioresponsive and multifunctional approach to wound care. They promote epithelialization, ensure a balanced cytokine environment, counteract microbial resistance, and facilitate the development of granulation tissue. Beyond their diverse mechanisms and safety profile, there remains a compelling rationale for further exploration of these innovative products in both translational and clinical research [138,139].

## 6. Comparative Analysis: Probiotic Nanoformulations vs. Conventional Therapies

Probiotic nano-formulations represent a notable advancement over conventional wound care methods, such as antibiotics and silver-based dressings. Traditional treatments often struggle with issues like insufficient biofilm penetration, limited biocompatibility, and rising antimicrobial resistance. In contrast, nanocarriers loaded with probiotics provide antimicrobial, anti-inflammatory, and regenerative benefits while preserving microbial balance and minimizing toxicity [140]. Numerous experimental studies demonstrate the superior efficacy of probiotic nano-formulations. For example, in a diabetic wound model using mice, chitosan nanogels containing *L. rhamnosus* achieved 89% wound closure within 10 days, compared to 62% in the neomycin group, along with a 2-log reduction in microbial load and significantly decreased TNF-α levels. Similarly, liposomal *L. acidophilus* enhanced epithelial regeneration and fibroblast activity while significantly reducing IL-6 levels compared to silver sulfadiazine in thermal wounds [141].

The nanocarriers employed in these systems, such as PLGA, alginate, and chitosan, are biodegradable and approved for medical use, ensuring they meet regulatory standards. Probiotics work not only by directly inhibiting pathogens but also by modulating the immune system. They encourage macrophage polarization towards an M2 phenotype, inhibit NF-κB signaling, and increase the production of growth factors like VEGF and TGF-β1—actions that antibiotics do not perform [142]. These varied mechanisms offer comprehensive support for healing in complex wound environments [85]. Consequently, given their superior efficacy, broader biological functions, and enhanced safety, probiotic nanoformulations present a compelling alternative to traditional wound care. As antibiotic resistance rises and biofilm-related infections continue, incorporating probiotic nano-therapy into clinical practice could transform wound management paradigms in the next decade, as outlined in the comparative analysis shown in (Table 2).

## 7. Challenges and Considerations in Probiotic Nanotechnology for Wound Healing

Although probiotic nanoformulations have the potential to enhance wound care, a number of challenges need to be addressed before their widespread application in clinical settings can occur. Making sure probiotics stay alive and metabolically active throughout the encapsulation and storage processes is the essential concern. Probiotics are encapsulated using techniques including spray drying, freeze-drying, or nanoprecipitation; nevertheless, shear stress, heat, and drying might reduce viability [152,153]. Encapsulation using chitosan-alginate has shown that over 80% of probiotics remain viable at four degrees Celsius for up to three months. However, certain strains experience a significant reduction in metabolic activity when exposed to ambient humidity [154].

A second significant challenge involves scalability and cost. While probiotic formulations at the laboratory level show substantial clinical benefits, expanding to commercial production necessitates further technical and economic considerations. Ensuring safety and consistency in nanoparticle size and bioactivity requires an infrastructure with quality control adhering to GMP (Good Manufacturing Practice) standards [155]. Economic analysis indicated that although probiotic dressings could potentially reduce long-term clinical costs, their initial expense remains 1.5 to 2 times higher than standard treatments [4]. Furthermore, obtaining regulatory approval for probiotic nanomedicine remains difficult. Unlike pharmaceuticals, probiotics are sometimes classified as food supplements or biologics, leading to regulatory inconsistencies across different countries. The integration of nanotechnology adds complexity to classification, safety evaluation, and approval processes. A 2022 report by the European Medicines Agency (EMA) highlighted the necessity for a unified regulatory framework to manage combined formulations involving both microbial and nanomaterial components. Additionally, preclinical studies should address the interaction between the host and probiotics, as well as the long-term biocompatibility of the nanomaterial [156].

Recent work [157] further emphasizes the importance of lyo-protectants and stabilizing agents in preserving probiotic viability during nano-delivery formulation. In various PEO-based nanofiber systems, species-specific efficacy of excipients was observed: sucrose best preserved *Lactobacillus jensenii*, while dextran and trehalose were most protective for *Staphylococcus* and *Stenotrophomonas* isolates. Notably, fully amorphous trehalose remained effective over 24 weeks, whereas semi-crystalline stabilizers (e.g., mannitol and glucose) showed reduced viability or even detrimental effects due to crystallization. These findings highlight that the choice and solid-state behavior of lyo-protectants is pivotal to maintaining long-term probiotic survival in wound care nano-formulations.

Ensuring stability and shelf life is crucial for probiotic-based nanotherapeutics, particularly in environments lacking cold chain distribution. Studies indicate that spray-dried nano-capsules using trehalose or skim milk as cryoprotectants exhibit superior thermal stability compared to in vitro experiments. However, when stored in standard packaging, they retain only 30–40% viability after 6 months at 25 °C. This reduction in viability not only diminishes probiotic counts but also affects bio-efficacy, potentially limiting therapeutic effectiveness [158,159]. As of this summer, ongoing research is investigating the use of oxygen scavengers and smart packaging to enhance shelf stability, though this might affect functionality.

It is essential to examine immunological risks as well. While probiotics are generally recognized as safe (GRAS), the potential for dysbiosis or negative immune reactions, particularly in immunocompromised individuals, should not be ignored. Instances of bacteremia and fungemia have been occasionally documented, mainly in patients undergoing live microbial therapy. Nanoformulations might further influence host responses by changing microbial adhesion characteristics or translocation behavior [160,161]. A study on mice revealed mild lymphocytic infiltration and increased IL-17 expression in wounds treated with *L. rhamnosus* nanofibers. These results underscore the importance of conducting individual risk-benefit analyses and evaluating product-specific toxicity [162].

## 8. Future Perspectives

Probiotic nanotechnology holds the potential to transform wound health care, especially in the areas of precision medicine and advanced therapeutics. The future of probiotic nanotechnology is promising, with the development of microbiome-targeted approaches to customize wound care [163]. Chronic wounds can vary depending on the bacterial composition of the host’s microbiota. By employing next-generation sequencing and microbiome profiling, future probiotic formulations could be customized to the specific local microflora, aiming to selectively diminish dysbiotic bacteria and restore microbial balance in infected wounds [164]. A 2023 review highlighted the introduction of strain-specific and site-specific probiotic nano-formulations to achieve better outcomes with fewer unintended effects. The integration of biosensing technologies into probiotic delivery systems is rapidly advancing. Smart wound dressings equipped with biosensors can detect changes in glucose, pH, reactive oxygen species, or pro-inflammatory cytokines and respond accordingly. These adaptive systems can release probiotics or their metabolites in a controlled manner, both temporally and spatially, enhancing therapeutic effectiveness while avoiding ineffective dosing [165].

The combination of artificial intelligence (AI) with probiotic nanomedicine could significantly impact chronic wound management. AI-driven platforms can analyze imaging, sensor, and microbiome data to forecast wound progression, recommend optimal treatment strategies, and evaluate probiotic effectiveness in real-time. Initial clinical models using machine-learning algorithms have shown about 92% accuracy in identifying healing trajectories. However, to integrate these findings into clinical practice, controlled trials, thorough validation, and regulatory alignment are necessary [166,167]. To evaluate the safety and effectiveness of probiotic nano-formulations for different wound types, such as diabetic foot ulcers, pressure ulcers, and burn injuries, several early-phase human trials are now being conducted. Nonetheless, widespread clinical adoption will require addressing challenges related to individual microbiome variability, the long-term safety of nanocarriers, and the complex approval process for live-biologic nanomedicines. Regulatory bodies are beginning to develop hybrid pathways to evaluate both microbial and nanomaterial components, with standard protocols still under discussion [168]. In the near future, we can expect the convergence of biotechnology, material science, and digital health to create multifunctional programmable probiotic systems capable of responding to wound dynamics in real-time. Next generation wound therapies will not only treat infections or inflammation but will also have the capability to diagnose, respond, and regenerate in real-time.

## 9. Conclusions

Probiotic nanotechnology signifies a groundbreaking development in chronic wound management by combining the advantages of live microbial therapy with nanocarrier-based delivery systems. This focused strategy tackles the complex issues of chronic wounds, including microbial biofilms, oxidative stress, inflammation, and hindered tissue regeneration. Nanoformulations such as liposomes, chitosan nanoparticles, microneedles, and ROS/glucose-responsive hydrogels improve probiotic stability, maintain viability, and allow for targeted, controlled release of bioactive substances. Probiotic strains like *L. plantarum*, *L. rhamnosus*, and *B. subtilis* have shown significant preclinical and clinical success, such as faster epithelialization, improved collagen deposition, biofilm disruption, and immune modulation through macrophage polarization and cytokine regulation. Additionally, advanced platforms featuring extracellular vesicles, postbiotics, and biosensor-integrated dressings indicate the rise of intelligent, adaptive wound therapies. When combined with AI-driven wound monitoring and microbiome profiling, these systems can provide personalized, precision-based care. Nonetheless, challenges persist in ensuring product stability, regulatory clarity, manufacturing scalability, and long-term biocompatibility. Overcoming these obstacles through standardized protocols, comprehensive clinical trials, and unified approval processes will be essential. Overall, probiotic nano-formulations offer tremendous potential to revolutionize wound care, providing a bio-responsive, cost-effective, and safer alternative to traditional antimicrobial treatments in an era of increasing antibiotic resistance.

## Figures and Tables

**Table 1:** Preclinical and Clinical Studies on Probiotics in Wound Healing.

Study Model	Probiotic Strain(s)	Delivery Route	Mechanism(s)	Key Outcome(s)	Ref.
Wistar rats (excision model)	*Lactobacillus acidophilus* + *B. bifidum*	Oral	Immunomodulation, cytokine regulation	↑ IL-10, ↓ TNF-α; 35% faster epithelialization by day 10	[74]
Diabetic mice (streptozotocin)	*L. plantarum*	Topical hydrogel	Angiogenesis, MMP regulation	↑ VEGF, ↓ MMP-9; accelerated granulation and collagen organization	[75]
Human clinical study (n = 30)	*Ligilactobacillus salivarius* (formerly known as *Lactobacillus salivarius*)	Oral (capsule)	Microbiota modulation, systemic inflammation reduction	Improved wound closure scores; ↓ CRP and IL-6 levels	[76]
Mice (burn model)	*Lactobacillus delbrueckii* subsp. *Bulgaricus*	Topical cream	ROS scavenging, AMP production	↓ MDA levels; ↑ catalase and SOD activity; reduced wound infection	[77]
Diabetic foot ulcer patients	Synbiotic (*L. casei* + Inulin)	Oral (capsule)	Gut-skin axis modulation, glycemic control	↓ HbA1c and pro-inflammatory markers; ↑ wound healing index vs. placebo	[78]

↑ indicates an upregulation or increase in the biomarker or cytokine level in response to treatment, suggesting enhanced biological activity. ↓ indicates a downregulation or decrease, signifying suppression of a specific inflammatory mediator.

**Table 2:** Comparative insights: Probiotic nanoformulations vs. Conventional Wound Therapies.

Parameter	Conventional Therapies	Probiotic Nanoformulations	Ref.
Mechanism of Action	Primarily antimicrobial; limited host interaction	Antimicrobial + immunomodulatory + antioxidant	[143]
Biofilm Penetration	Limited efficacy due to structural resistance	Penetrates biofilms; secretes EPS-disrupting enzymes	[144]
Impact on Microbiome	Disrupts both pathogenic and commensal flora	Promotes microbial balance and skin homeostasis	[145]
Healing Efficacy (Preclinical)	Slower wound closure; high inflammation	Faster epithelialization, angiogenesis, and collagen deposition	[146]
Healing Efficacy (Clinical)	Variable, often limited in chronic wounds	40–50% faster wound area reduction; improved pain and inflammation	[147]
Resistance Potential	High; overuse leads to MDR pathogens	Low; does not promote antibiotic resistance	[148]
Cost-Effectiveness	Initially low cost but high recurrence and complication rate	Higher upfront cost, but reduced treatment time and recurrence	[149]
Regulatory Status	Well-established	Emerging; requires dual-level evaluation (probiotic + nanocarrier)	[150]
Adverse Effects	Possible cytotoxicity, allergic reactions, microbiome loss	Minimal adverse effects in trials; high biocompatibility	[151]

## Data Availability

No new data were created or analyzed in this study. Data sharing is not applicable to this article.

## Abbreviations

The following abbreviations are used in this manuscript:

DFUs	diabetic foot ulcers
VLUs	venous leg ulcers
AMR	antimicrobial resistance
MDR	multi-drug resistance
TNF-α	tumor necrosis factor-alpha
IL-1β	interleukin-1 beta
MMPs	matrix metalloproteinases
ROS	reactive oxygen species
AGEs	advanced glycation end-products
TLR	toll-like receptor
AMPs	antimicrobial peptides
VEGF	vascular endothelial growth factor
TGF-β1	transforming growth factor-beta 1
EVs	Extracellular Vesicles
CRP	C-Reactive Protein
SOD	Superoxide Dismutase
MDA	Malondialdehyde
PCL	Polycaprolactone
PVA	polyvinyl alcohol
MN	Microneedle
PLGA	Poly(lactic-*co*-glycolic acid)
bFGF	basic fibroblast growth factor
ECM	extracellular matrix
SLNs	solid lipid nanoparticles
MOF	Metal-organic frameworks
PAMAM	poly(amidoamine)
EMA	European Medicines Agency
